# Comparative Evaluation of MRSA Nasal Colonization Epidemiology in the Urban and Rural Secondary School Community of Kurdistan, Iraq

**DOI:** 10.1371/journal.pone.0124920

**Published:** 2015-05-01

**Authors:** Nawfal R. Hussein, Zarrin Basharat, Ary H. Muhammed, Samim A. Al-Dabbagh

**Affiliations:** 1 Department of Internal Medicine, School of Medicine, Faculty of Medical Sciences, University of Duhok, Kurdistan Region, Iraq; 2 Microbiology and Biotechnology Research Lab, Department of Environmental Sciences, Fatima Jinnah Women University, Rawalpindi, Pakistan; 3 Department of Community Medicine, School of Medicine, Faculty of Medical Sciences, University of Duhok, Kurdistan Region, Iraq; 4 Department of Family & Community Medicine, School of Medicine, Faculty of Medical Sciences, University of Duhok, Kurdistan Region, Iraq; Ross University School of Veterinary Medicine, SAINT KITTS AND NEVIS

## Abstract

**Background:**

To study the nasal carriage rate of *Staphylococcus aureus* (*S*. *aureus*) (including methicillin-resistant strains) in secondary school community of the urban and rural districts of the Kurdistan region of Iraq, a cross-sectional population based survey was carried out in the city Duhok and rural areas of Amedya, Akre and Zakho.

**Methods:**

Nasal swabs were obtained from nostrils of 509 students aged 14-23 years. Resistance to methicillin was assessed by Kirby-Bauer disk diffusion and agar dilution assay. Vancomycin sensitivity was also tested on Muller-Hinton agar.

**Results:**

It was found that the frequency of overall *S*. *aureus* nasal carriage (SANC) was 17.75% (90/509, CI_95_, 14.58–21.42%). In urban areas, the carriage rate was 20.59% (49/239, CI_95_, 15.64–26.29%), whereas it was 15.24% (41/270, CI_95_, 11.17–20.10%) in rural districts. The frequency of methicillin-resistant *S*. *aureus* (MRSA) among the isolated strains was found to be 2.04% (1/49) and 21.95% (9/41) in urban and rural areas respectively. It was found that in urban residents, the odd ratio (OR) of acquiring SANC was 1.44 (CI_95_, 0.91-2.27%) and risk ratio (RR) was at least 1.35 (CI_95_, 0.92-1.96%) while OR decreased to 0.12 (CI_95_, 0.01-0.96%) for MRSA carriage. Hence, the *S*. *aureus* carriage rate was higher in urban districts compared to rural areas while more MRSA were found in rural areas compared to urban districts. All studied strains were sensitive to vancomycin.

**Conclusion:**

This study provided baseline information for *S*. *aureus* nasal colonization in the region. Also, it showed that living in rural areas increased the odds of MRSA colonization. More attention should be paid to control MRSA colonization in rural communities.

## Introduction

*Staphylococcus aureus* is a common cause of numerous local and disseminated infections of the skin, respiratory system, bloodstream, urinary tract and soft tissues [1]. Different species of Staphylococci can infect humans but *S*. *aureus* accounts for almost a quarter of such infections. This is due to its virulence potential and frequent colonization in humans and livestock. Infections can be either community or healthcare facility acquired and the severity of these infections is dependent upon the strain virulence and strength of the host immune system [2]. Community acquired MRSA (CA-MRSA) infection has become a recognized public health concern and if unchecked, feared to reach epidemic proportions [3]. Virulence characters like toxin production, expression of antibody inactivating cell-surface proteins and high transmission rates are the trademarks of CA-MRSA strains. Healthcare-associated methicillin-resistant *S*. *aureus* (HA-MRSA) can cause infections within the healthcare facilities such as surgical site infections, life threatening bloodstream infections and septic shock [4]. MRSA colonization is a major factor as colonized individuals are at augmented risk of attaining the infections [5]. Nose is the main colonization site of the human body [6], with the possibility of occurrence of colonization in extra-nasal sites such as in the perineum [7]. Conventional screening of MRSA is performed by using selective and differential agar media and the results can be obtained within 18 to 48 hours. Faster detection can be achieved by using polymerase-chain reaction (PCR) based assays [8], [9]. The *mecA* gene is a robust marker for the accurate detection of MRSA isolates [10]. This gene is found on 21 to 60-kb staphylococcal chromosome cassette *mec* (SCC*mec*) conferring resistance to methicillin [11]. Almost eleven type of SCC*mec* elements (I–XI) have been identified to date. SCC*mec* elements carry similar characteristics and consist of a cassette chromosome recombinase for site-specific insertion [12–13].

MRSA colonization in subjects living in the city and the countryside has been studied in many countries such as Portugal [13], Bolivia, Peru [14], Ghana [15] and northern Vietnam [16]. To the best of our knowledge, such a study has not been performed in Iraq previously. Therefore, we conducted a study in urban and rural areas of Iraq to analyse differences in the carriage rate of *S*. *aureus* including MRSA.

## Materials and Methods

### Sample collection

More than five hundred students from secondary schools were included in this study. Samples were taken between January and June 2014, including subjects from both urban area of Duhok (depicted as C) (N = 239) and surrounding rural areas in three districts: Amede, Akre and Zakho (depicted collectively as R with N = 270). When we started the study, no previous report of the prevalence of nasal carriage of MRSA in this location had been conducted, therefore, a prevalence of 50% was chosen for calculating the sample size. With a confidence interval of 95% (Z = 1.96), margin of error of 0.05, design effect of 1 and expected response rate of 0.8, the sample size calculated was 480. However, during the time allocated for the study, 509 participants were recruited.

A random sample from secondary school students was taken in a multi-stage process. Firstly, the number of participants from each district was estimated according to the total number of students in secondary schools. It was roughly estimated to take a sample of 40 students from each visited school. Schools were then selected at random by a simple random sampling method and a class within the school was chosen by the same method. Within the class, all students, unless they had an exclusion criterion, were included in the study.

Because hospitalisation and admission to healthcare facilities increase the risk of MRSA colonization, we excluded every student with:

1. History of hospitalization, surgery, dialysis or residence in a long-term care facility within one year of the MRSA culture date.
2. The presence of an indwelling catheter or a percutaneous device at the time of culture.
3. Previous isolation of MRSA.

### Ethics statement

Informed written consent was obtained in the form of questionnaires from students older than eighteen years with permission from their parents/legal guardian. For students younger than eighteen years, written consent was recorded from guardians on behalf of subjects involved in the study. All information was anonymized before analysis [17]. This study and method of attaining consent was approved by Ethics committee in the University of Duhok, School of Medicine, Kurdistan Region, Iraq.

### Bacterial identification and antimicrobial susceptibility testing

Specimens were taken via the insertion of a sterile moistened swab in both nostrils to a depth of approximately 1 cm into the nostril and rotated five times. After collection, specimens were immediately transported to the lab for inoculation on the culture medium. Samples were directly inoculated onto mannitol salt agar plates and incubated at 35°C for 48 hours. *S*. *aureus* isolate identification was based on morphology, Gram’s stain property, coagulase test, catalase test and mannitol salt agar fermentation. Antimicrobial susceptibility testing to oxacillin was carried out according to Clinical Laboratory Standards Institute (CLSI) recommendations [18] using Kirby-Bauer disk diffusion and agar dilution assay methods using Muller-Hinton agar (Oxoid Limited, Hampshire, England). BHI agar plates supplemented with 6 μg/ml vancomycin were used for testing of strains for vancomycin resistance. After adjusting the bacterial suspension to the concentration of 0.5 McFarland, 10μl inoculum was spread on the agar plate (final concentration = 10^6^ CFU/ml). In addition to this, agar dilution assay was used to determine vancomycin minimum inhibitory concentration (MIC). Strains for which vancomycin MICs were less than 2 μg/mL were classified as sensitive, MIC of 2–8 μg/mL were considered as vancomycin-intermediate, and strains for which vancomycin MICs were ≥16 μg/mL were considered as vancomycin-resistant. All experiments were repeated thrice.

### DNA extraction and mecA PCR

DNA was extracted from *S*. *aureus* isolates using the Qiagen DNA Purification kit as per manufacturer’s instructions (Qiagen). All methicillin-resistant isolates were examined for the existence of the *mecA* gene as previously described [19]. The presence of the *mecA* gene was confirmed by PCR amplification as described by Murakami *et al*. [20]. Thermal cycling for amplifying *mecA* was 95°C for 30 s, 58°C for 1 min and 72°C for 2 min, for a total of 35 cycles. PCR amplification of *mecA* used previously described primers (MR1: GTGGAATTGGCCAATACAGG and MR2: TAGGTTCTGCAGTACCGGAT) that can amplify upto 1399 base pair fragments specific for *mecA* gene [21]. Amplification of the gene started with an initial denaturation at 95°C for 60 s and a final elongation step of 5 min at 72°C. Reactions were performed in 25 μl volume containing 1 μl of genomic DNA, 1 μl primer, 0.5 μl of Taq DNA polymerase, 0.5 μl dNTP, and 2.5 10X PCR buffer. Then 5μl of the PCR products were electrophoresed in 1.5% agarose gels for 40 minutes at 80 V in 1X TAE buffer. All gels were stained with ethidium bromide (1 mg/l) and visualized under UV light. 100 bp DNA ladder (Gibco, Paisley, UK) was used as a size marker (M) in all gels.

### Statistical analysis

Study population characteristics were defined by descriptive analysis. Place of residence was stratified into two main regions, Rural and Urban, age into 10 and sex into two categories. Prevalence of nasal *S*. *aureus* carriage with its 95% confidence interval (CI_95_) was described. Univariate analysis was conducted to assess association of rural and city residence with nasal *S*. *aureus* carriage and methicillin resistance. The strength of associations was based on crude and adjusted odds ratios (OR) and CI_95_. Statistical analysis was carried out with with EPI Info 7.1.4 software (http://wwwn.cdc.gov/epiinfo/7/) and p <0.05 values were considered as significant.

## Results and Discussion

The role of *S*. *aureus* in infections occurring in the community and its ability to spread from person to person is well established. Numerous studies reported the importance of MRSA colonization and it was shown that colonization with such a strain increased the likelihood of invasive disease [22], [23]. Also, the transmission of MRSA was shown to occur in relatively ‘closed’ populations such as in schools, day care centres or sport facilities [24]. Because of the global increase of MRSA carriage rates, the infection with such a strain has increased. For example, an increment in MRSA infection from 19.5% to 31.9% was observed in Canada and also it was found that 70% of skin and skin structure infections was caused by MRSA [25]. Such an increase might be the herald of an epidemic of MRSA infection that can involve skin and soft tissue infections, necrotizing pneumonia and severe sepsis especially in resource-poor regions [3].

The purpose of this project was to study the *S*. *aureus* and MRSA carriage rate in young people attending secondary schools in urban and rural areas in Kurdistan region, Iraq. In studied secondary school populace of city centre in Duhok and rural areas of the three districts (Akre, Zakho and Amedya), the highest frequency of SANC was among the students aged sixteen 27.77% (25/90) while 0% (0/90) was observed in students aged fourteen (Table 1). In agreement with our previous project studying the carriage rate in urban areas [26], the frequency of SANC was 20.59% (49/239, CI_95_, 15.64–26.29%) in urban territories while it was found to be 15.24% (41/269, CI_95_, 11.17–20.10%) in the rural area. Among the *S*. *aureus* isolates, MRSA strains were higher in rural (22.50%) (9/41, CI_95_, 10.84–38.45%) than urban areas (2.08%) (1/49, CI_95_, 0.05–11.07%). Since 1990s, many countries have experienced rising up in the prevalence of MRSA [27]. In previously reported studies, prevalence of MRSA in the community ranged from very low in Netherlands to around 47.15% in Nigeria [28]. In the present study, MRSA carriage rate was studied in the Iraqi population, which showed that carriage frequency was 0.42% (1/239, CI_95_, 0.01–2.31%) in urban area, 3.33% (9/270, CI_95_, 1.54–6.23%) in rural districts and 1.96% (10/509, CI_95_, 1.00–3.7%) for the entire tested community. Current study revealed that MRSA colonization rate among the entire studied population was almost equivalent to that of Taiwan (1.9%) [29], higher than Ohio State (0%) [30] and much lower than that reported from Pakistan (24%) [26].

**Table 1:** Frequency distribution of ‘age’ with ‘*S*. *aureus’* colonized subjects among the studied secondary school student population.

Urban area					Rural districts			
Age	Frequency	Percent (%)	CI_95_ Lower (%)	CI_95_ Upper (%)	Frequency	Percent (%)	CI_95_ Lower (%)	CI_95_ Upper (%)
14	0	0.00	0.00	7.25	0	0.00	0.00	8.60
15	7	14.29	5.94	27.24	9	21.95	14.38	37.61
16	11	22.45	11.77	36.62	14	34.15	23.72	50.59
17	10	20.41	10.24	34.34	6	14.63	7.70	29.17
18	9	18.37	8.76	32.02	3	7.32	2.70	19.92
19	6	12.24	4.63	24.77	3	7.32	2.70	19.92
20	2	4.08	0.50	13.98	2	4.88	1.33	16.53
21	2	4.08	0.50	13.98	2	4.88	1.33	16.53
22	1	2.04	0.05	10.85	2	4.88	1.33	16.53
23	1	2.04	0.05	10.85	0	0.00	0.00	8.60
TOTAL	N = **49**	**100.00**			**N = 41**	**100.00**		

It was found that based on the place of dwelling in Iraq, the OR of acquiring SANC was 1.44 (CI_95_, 0.91–2.27%) and risk ratio (RR) was at least 1.35 (CI_95_, 0.92–1.96%) for urban residents while OR decreased to 0.12 (CI_95_, 0.01–0.96%) for MRSA carriage. Hence, the *S*. *aureus* carriage rate was higher in urban districts but with less MRSA strains in comparison to rural areas, where the *S*. *aureus* carriage was less but with higher MRSA strains. Our data emphasizes the importance of appropriate infection control measures such as the use of disinfectants, frequent hand washing and application of proper sanitary measures. Such measures should be imposed in school communities especially around Akre, Amedya and Zakho to decrease the likelihood of MRSA nasal carriage in the population and prevent the outbreak of MRSA infections. Regular follow-up studies of MRSA carriage in school students/monitoring of other factors, such as the similarity or difference of infecting clones, multiple site sampling, history of skin or soft tissue infection, sharing of towels/sports equipment at school, household member working in health care facility and regular visits to health care facility, need to be undertaken. Such studies could be helpful in future analysis of the trends of MRSA carriage rates and infections. Also, the information would be useful to estimate the proportion of SANC in the urban and rural population.

The frequency of SANC in females was 20.91% (23/110, CI_95_, 13.74–29.70%) in urban as compared to 17.89% (22/123, CI_95_, 11.56–25.82%) in rural areas and that of males was 20.31% (26/128, CI_95_, 13.72–28.33%) in urban as compared to 13.01% (19/146, CI_95_, 8.02–19.57%) in rural districts (Table 2). In addition, the overall frequency of SANC and MRSA in female was found to be 19.23% (45/234) and 2.56% (6/234) respectively. In males, the SANC and MRSA carriage rate was found to be 16.36% (45/275) and 1.45% (4/275) respectively. Based on gender, OR of acquiring SANC was 1.2 (CI_95_, 0.77–1.91%) and RR was 1.17 (CI_95_, 0.80–1.70%) for females. Same trend was found in females for MRSA carriage as the OR and RR was found to be 1.78 (CI_95_, 0.49–6.39%) and 1.76 (CI_95_, 0.50–6.17%), respectively. This indicated that SANC and MRSA carriage was considerably gender linked in the secondary school population of Iraq. The basis of this gender based carriage needs to be probed further, especially in regard to habits influencing hygiene of both males and females. This might shed light on the cause of carriage and risk variation among individuals of both genders.

**Table 2:** Univariate analysis of studied variables in the analyzed secondary school student population of urban area “Duhok” and rural areas of ‘Akre’, ‘Amedya’ and ‘Zakho’ districts.

Region	Variable	Category	Frequency	Percent (%)	Cum. Percent (%)	CI_95_ Lower (%)	CI_95_ Upper (%)	Region	Variable	Category	Frequency	Percent (%)	Cum. Percent (%)	CI_95_ Lower (%)	CI_95_ Upper (%)
**Urban**	**Sex**	Female	23	46.94	46.94	32.53	61.73	**Rural**	**Sex**	Female	22	53.66	53.66	37.42	69.34
		Male	26	53.06	100.00	38.27	67.47			Male	19	46.34	100.00	30.66	62.58
	***S*. *areus* colonization**	No	189	79.41	79.41	73.71	84.36		***S*. *aureus* colonization**	No	228	84.76	84.76	79.90	88.83
		Yes	49	20.59	100.00	15.64	26.29			Yes	41	15.24	100.00	11.17	20.10
	**MRSA**	No	47	97.92	97.92	88.93	99.95		**MRSA**	No	31	77.50	77.50	61.55	89.16
		Yes	1	2.08	100	0.05	11.07			Yes	9	22.50	100.00	10.84	38.45

Minimum inhibitory concentration of oxacillin was 16 μg/ml (7 strains), 32 μg/ml (2 strains) and 64 μg/ml (1 strain). Resistance to the penicillinase-stable antibiotics such as oxacillin is due to the expression of a new penicillin binding protein, PBP2a (PBP2′), which is encoded by the *mecA* gene. All our MRSA strains reported in this study typed positive for *mecA* gene. Borderline oxacillin-resistant *S*. *aureus* (BORSA) can sometimes be confused with MRSA because of similar clinical signs and symptoms and overlapping oxacillin MICs (2–8 μg/ml for BORSA and 4–64 μg/ml for MRSA) [31]. The mechanism of resistance exhibited by BORSA includes excessive penicillin production, plasmid mediated inducible methicillinase, or point mutations of penicillin-binding proteins [31]. None of our *S*. *aureus* strains was BORSA due to the high MIC of our strains and the presence of *mecA* gene.

Vancomycin resistant *S*. *aureus* (VRSA) was first reported in the United States in 2002. Since then, limited reports have been published about the isolation of VRSA from clinical specimens from around the world. In Iran, (a neighboring country to Iraq) only 24 VRSA strains have been isolated so far [32]. To the best of our knowledge, no vancomycin resistant strain has been reported in Iraq. In this report, none of the methicillin resistance strains showed resistance to vancomycin. Therefore, vamcomycin can be regarded as one of the options for the empirical treatment of infection caused by MRSA.

## Conclusion

It was seen that the *S*. *aureus* carriage rate was higher in urban districts but with less MRSA strains, in comparison to rural areas, where the *S*. *aureus* carriage was less but with higher MRSA strains. The carriage rate reached its peak at the age of 16. This study is the first to compare the MRSA carriage rate between the urban and rural communities in Iraq. Therefore, it may serve as much-needed baseline information for the general public (parents), government, school bodies or agencies for ensuring good health of Iraqi school children by introducing and implementing effective measures to decrease *Staphylococcus* colonization.

## Supporting Information

S1 Dataset (XLSX)
